# Presynaptic mechanisms of impaired long term potentiation of lateral entorhinal cortex input to CA1 in the PS19 mouse model of tauopathy

**DOI:** 10.1002/alz.086703

**Published:** 2025-01-03

**Authors:** Mohankumar Thangavel, Chengju Tian, Isabel Reyes, Arjun V. Masurkar

**Affiliations:** ^1^ New York University Grossman School of Medicine, New York, NY USA; ^2^ NYU Grossman School of Medicine, New York, NY USA

## Abstract

**Background:**

How tauopathy disrupts direct entorhinal cortex (EC) inputs to CA1 and their plasticity is understudied, despite its critical role in memory. Moreover, dysfunction of lateral EC (LEC) input is less clear, despite its relevance to early Alzheimer’s disease pathogenesis. Here we examined how tau impacts long‐term potentiation (LTP) of LEC→CA1 input in a transgenic model of tauopathy.

**Method:**

Acute hippocampal slices were prepared from PS19 and WT mice (8‐10 mo. old, M/F) in which LEC had been injected with an AAV expressing channelrhodopsin ChR2. Ex vivo recordings were achieved with blue LED light to excite LEC axons and an extracellular field electrode in *stratum lacunosum moleculare* (SLM) of distal CA1 to record field responses. Input‐output curves were established using 25‐100% LED power for field postsynaptic excitatory potentials (fEPSPs) with inhibition blocked (GABAA: 2mm SR95531, GABAB: 1mm CGP55845). LTP of fEPSPs was elicited after a 10” baseline using an LED theta burst stimulation (TBS). Paired pulse ratio (PPR) of fEPSPs was elicited with LED stimulation of 2 pulses at 20Hz. Fluorescence immunohistochemistry (IHC) was performed in separate mice using antibodies to N‐type calcium channels.

**Result:**

All results are based on n = 4‐8/sex/genotype. The LEC fEPSP was of reduced magnitude in female PS19 versus WT mice, while unchanged across genotype in male mice. TBS induced a late‐onset LTP (147% at 45”) of the LEC fEPSP in female WT mice, which was significantly reduced in female PS19 mice (p = 0.0051). There was no LTP reduction in male mice. LTP in female WT mice was associated with a PPR reduction that was reversed by the addition of the N‐type calcium channel blocker w‐conotoxin GVIA, all of which did not occur in PS19 mice (p<0.0001). PPR was appropriately reduced in male mice across genotype. IHC of N‐type calcium channels showed reduced fluorescence in the SLM of female PS19 mice versus WT.

**Conclusion:**

In PS19 mice, the impact of tau on LEC→CA1 input is sex‐dependent, with reduced LEC‐driven excitation and LTP only in female mice. A reduction in presynaptic N‐type calcium channels in PS19 mice may serve as a presynaptic mechanism for the reduced plasticity.

